# Anti-SARS-CoV-2 virus antibody levels in convalescent plasma of six donors who have recovered from COVID-19

**DOI:** 10.18632/aging.103102

**Published:** 2020-04-22

**Authors:** Libo Zhang, Rongrong Pang, Xiang Xue, Jingjing Bao, Sheng Ye, Yudong Dai, Yishan Zheng, Qiang Fu, Zhiliang Hu, Yongxiang Yi

**Affiliations:** 1 Department of Laboratory Medicine, Nanjing Red Cross Blood Center, Nanjing 210003, Jiangsu, China; 2 Department of Biochemistry and Molecular Biology, University of New Mexico, Albuquerque, NM 87131, USA; 3 Department of Apheresis, Nanjing Red Cross Blood Center, Nanjing 210003, Jiangsu, China; 4 Department of Blood Management, Administrative Office, Nanjing Red Cross Blood Center, Nanjing 210003, Jiangsu, China; 5 Department of Critical Medicine, The Second Hospital of Nanjing, Nanjing University of Chinese Medicine, Nanjing 210003, Jiangsu, China; 6 Nanjing Infectious Disease Center, The Second Hospital of Nanjing, Nanjing University of Chinese Medicine, Nanjing 210003, Jiangsu, China; 7 School of Public Health, Nanjing Medical University, Nanjing 211166, Jiangsu, China

**Keywords:** coronavirus disease 2019, COVID-19, convalescent plasma, SARS-CoV-2 virus, anti-SARS-CoV-2 antibodies, plasma donation

## Abstract

Background: Anti-SARS-CoV-2 virus antibody levels in convalescent plasma (CP), which may be useful in severe Anti-SARS-CoV-2 virus infections, have been rarely reported.

Results: A total of eight donors were considered for enrollment; two of them were excluded because of ineligible routine check. Of the six remaining participants, five samples were tested weakly positive by the IgM ELISA. Meanwhile, high titers of IgG were observed in five samples. The patient treated with CP did not require mechanical ventilation 11 days after plasma transfusion, and was then transferred to a general ward.

Conclusions: Our serological findings in convalescent plasma from recovered patients may help facilitate understanding of the SARS-CoV-2 infection and establish CP donor screening protocol in COVID-19 outbreak.

Methods: Anti-SARS-CoV-2 antibodies including IgM and IgG were measured by two enzyme-linked immunosorbent assays (ELISA) in convalescent plasma from six donors who have recovered from coronavirus disease 2019 (COVID-19) in Nanjing, China. CP was also utilized for the treatment of one severe COVID-19 patient.

## INTRODUCTION

By late 2019 the outbreak of coronavirus disease 2019 (COVID-19) was unchecked in China [1, 2]. Apart from supportive care, specific drugs for this disease are still being researched [3, 4]. The absence of efficacy-proven antiviral treatment has led to attempts to treat severe SARS-CoV-2 infection with convalescent plasma containing SARS-CoV-2 specific antibodies from recovery patients-a precedent established with pathogen-specific immunoglobulin therapy for Ebola virus disease, influenza, severe acute respiratory syndrome, and severe fever and thrombocytopenia syndrome [5–8].

Previous reports on other viral infections have suggested that convalescent plasma with higher antibody levels may have great effect on virus load [9, 10], and our study was designed to test anti-SARS-CoV-2 virus antibody levels to select those with high titers, desiring a meaningful serologic response after CP infusion.

In accordance with CP infusion therapeutics guidelines approved by the National Health Commission of People's Republic of China, we used ELISA to screen for anti-SARS-CoV-2 IgM and IgG. In this report, we present our preliminary findings of anti-SARS-CoV-2 antibody levels in convalescent plasma obtained from six donors and clinical effects of one case treated with CP in Nanjing, China.

## RESULTS

### Characteristics of the six CP donors

We recruited a total of six donors including four males and two females, aged from 30 to 50 years old, with laboratory confirmed SARS-CoV-2 infection during the COVID-19 outbreak and the subsequent recovery certificated by two consecutively negative SARS-CoV-2 PCR assays and resolution of clinical symptoms. All the donors had fever and cough during the course of COVID-19. None of the donors were currently smoking. Donor D had a history of brain surgery due to a benign tumor. The other five donors did not have any underlying comorbidities. The baseline blood examinations of the donors, when they were admitted to the hospital due to COVID-19, were summarized in Table 1. At the time of admission, two donors had lymphocytopenia (lymphocyte counts<0.8×10^9^/L), one donor had increased alanine aminotransferase level (144 IU/L), one donor had elevated creatine kinase level (490 U/L), three donors had abnormal lactate dehydrogenase (ranged from 261 to 286 IU/L) and four donors had a C-reactive protein level of more than 10 mg/L (Table 1). Chest CT scans demonstrated bilateral pneumonia in all six donors.

**Table 1:** Baseline blood examinations of the six donors when they were admitted to the hospital due to COVID-19.

**Donor No.**	**Age, y/sex**	**WBC, ×10^9^/L**	**Lymphocyte counts,×10^9^/L**	**ALT, IU/L**	**Creatinine, μmol/L**	**CK, U/L**	**LDH, IU/L**	**Troponin I, ng/mL**	**D-dimer, μg/L**	**PT, s**	**Procalcitonin, ng/mL**	**IL-6**	**CRP, mg/L**
A	30/M	5.52	1.67	22.7	84	140	261	0.05	0.18	12	0.024	0.014	< 10.00
B	37/M	4.7	0.63	22.1	47	490	265	0.01	NA	12.4	0.039	0.055	63.77
C	45/F	3.42	1.41	28.1	43	34	141	0.05	0.53	11.9	0.013	0.006	16.09
D	42/M	5.65	0.71	12.5	64.5	39	223	0.009	0.19	13.0	0.076	0.084	21
E	32/M	4.32	1.46	16	57	60	188	0.25	0.26	12	0.410	0.031	< 10.00
F	50/F	4.06	0.99	144	38	47	286	0.06	0.19	10.1	0.013	0.031	12.4

WBC, white blood cell counts; ALT, alanine aminotransferase; CK, creatine kinase; LDH, lactate dehydrogenase; PT, prothrombin time; IL-6, interleukin 6; CRP, C-reactive protein; NA, not available.

During hospitalization, all donors were routinely given antiviral therapy with interferon-α (500 WU, twice a day, aerosol inhalation) and lopinavir/ritonavir (400/100mg, twice a day). Donor B, C, D, and E also received intravenous immunoglobulin. A 3-day course of corticosteroids (methylprednisolone 40 mg per day) was administered to donor B, D and F. None of donor needed mechanical ventilation or required to be transferred to the intensive care unit. The time from onset of symptoms to clearance of virus, defined as two consecutive negative nucleic acid tests from throat swab samples, were varied from 8 to 18 days. The donors were discharged after virus clearance and substantially improvement of their pneumonia.

Plasma samples were collected at times ranging from 29 to 46 days after symptom onset, and 13 to 27 days after their discharge, respectively (Table 2). At the time of blood donation, the donors were free of any symptom. The complete blood count, liver and renal function, lactate dehydrogenase, and C-reactive protein were within the normal range. The lymphocyte subsets counts were summarized in Table 3. All ABO types were involved in the study except AB type. Additionally, as part of the routine check, the donated plasma was confirmed free of hepatitis B and C virus, human immunodeficiency virus (HIV) and residual SARS-CoV-2 by RT-PCR and serologic negative for hepatitis B and C virus, HIV, and syphilis.

**Table 2:** Antibody levels of six donors recovered from COVID-19.

**Donor No.**	**Blood group**	**Days from symptom onset to plasma collecting**	**Days from discharge to plasma collecting**	**Anti-SARS-CoV-2 IgM levels (OD ratio)^a^**	**Anti-SARS-CoV-2 IgG levels (OD ratio)^a,b^**
A	A	29	13	1.47	7.58
B	O	36	17	1.22	6.59
C	B	37	23	1.55	7.84
D	A	46	27	2.01	3.92
E	O	40	22	1.95	7.52
F	A	39	27	5.63	8.36

^a^Negative controls and positive controls were included in every run.

^b^Serial tests were performed (Figure 1).

**Table 3:** Lymphocyte subsets counts of the six donors at the time of blood donation.

**Donor No.**	**Lymphocyte counts (cells/ul)**	**CD3+(%)**	**CD3+(cells/ul)**	**CD3+CD4+(%)**	**CD3+CD4+(cells/ul)**	**CD3+CD8+(%)**	**CD3+CD8+(cells/ul)**	**CD4/CD8 ratio**	**CD16+CD56+NK cells (%)**	**CD16+CD56+NK cells (cells/ul)**	**CD19+ (%)**	**CD19 (cells/ul)**
A	2619	63.23	1656	20	512	30	792	0.65	20.43	535	13.21	346
B	1970	67.36	1327	30	585	33	643	0.91	19.39	382	8.07	159
C	1690	67.81	1146	28	465	30	503	0.92	20.41	345	9.7	164
D	1796	70.99	1275	36	653	30	542	1.2	21.55	387	5.57	100
E	1841	57.47	1058	34	625	17	318	1.97	17.82	328	20.64	380
F	1645	80.3	1321	52	850	25	416	2.04	9.3	153	9.18	151

### Serological findings of anti-SARS-CoV-2 antibodies detected by ELISA

The anti-SARS-CoV-2 IgM antibody was weakly reactive (OD ratio from 1.22 to 2.01) for all donors except donor F, with a slightly higher OD ratio of 5.63, and IgG ELISA assay were also positive (OD ratio from 3.92 to 8.36) for all six donors who had IgM reactive plasma samples (Table 2).

**Figure 1:**
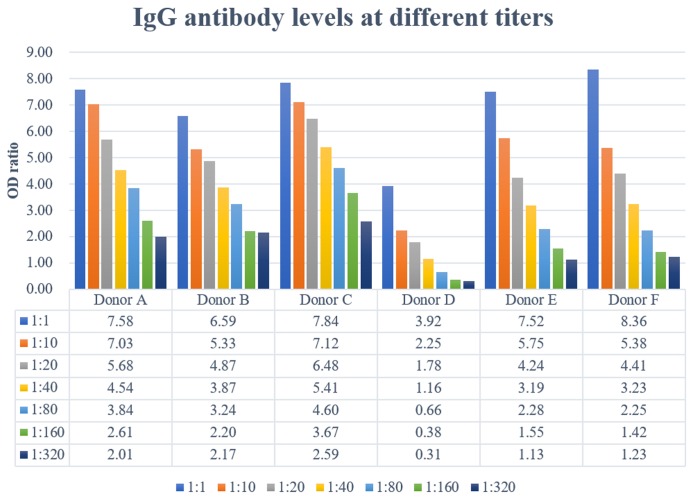
**Specific immunoglobulin IgG were titrated by semiquantitative ELISA.** Plasma IgG antibody titers ranged from 1:40 to >320.

All donors but one had high IgG titers (≥1:320) (Figure 1), meeting the criteria (≥1:160) sponsored by the National Health Commission. However, donor D had a low IgG titer (1:40) (Figure 1), therefore this donor was not considered as an eligible donor. This donor, a 42-year-old man had the longest duration (46 days) from symptom onset to plasma collection and he had the longest duration (19 days) of hospital stay. Also, this donor had the lowest CD19+ B-cell count as well as percentage in the lymphocyte subsets analysis (Table 3).

### Clinical utility of CP in a critically ill patient

The recipient for CP was a 64-year-old female. The patient was admitted to the hospital because of fever, fatigue, nausea and vomiting for 3 days, and was then confirmed of COVID-19. The underlying commodities included hypertension and diabetes. There was a fast progression of the clinical condition. On day 4 of hospitalization, the patient was transferred to Intensive Care Unit (ICU) and 1 week later received invasive mechanical ventilation. SARS-CoV-2 was undetectable from throat swab sample by nucleic acid test at the time of intubation. On day 17 of hospitalization, while the patient was still receiving invasive mechanical ventilation with a PaO2/FiO2 of 166 mmHg, she was given 200 mL CP from donor B. At the time of plasma transfusion, the lymphocyte count was 0.44×10^9^/L. Other blood examinations, including renal and liver function, prothrombin time, creatine kinase, lactate dehydrogenase and myocardial enzymes, did not significantly changed, although the D-dimer was increased (2.31mg/L). There was no transfusion related adverse event. Lymphocyte count remained below 0.5×10^9^/L for 1 week. The patient did not require mechanical ventilation 11 days after plasma transfusion, and was then transferred to a general ward.

## DISCUSSION

We reported the serological findings of SARS-CoV-2 infection in a CP donor population. Our preliminary findings suggest that recently recovered COVID-19 patients may be suitable potential donors, provided they meet other blood donation criteria.

Although our experience is limited in a few cases, a possibility could be suggested that, different from other viruses like MERS-CoV infection [11], antibody to SARS-CoV-2 in serum or plasma was frequently reactive by ELISA. All of the six donors showed positive IgM results, indicating that a negative result for IgM, a serologic marker which usually represents a recent or current infection [12–14], may not be suitable to be taken as a mandatory requirement for CP donor selection of limited availability of eligible potential donors in a COVID-19 outbreak. Of the six donors, only one donor had IgG titers of 1:40, which did not meet the criteria 1:160 recommended by the National Health Commission. Of note, compared with other donors, he experienced a severe disease, and had the longest duration from symptom onset to plasma collection, we suspected whether this phenomenon was related to his low CD19+ B-cell count or he had experienced a viral reactivation-an observation that requires further investigation.

However, due to limitations imposed by sample size, reactivity of ELISA tests may also be affected by the timing of plasma collection, severity of illness or corticosteroids administration. In addition, although this life-threatening disease appear to be under control following nationwide efforts and implementation of quarantine policy in China, but it is still developing in the other parts of the world. As yet no reference materials of anti-SARS-CoV-2 antibodies has been made available to evaluate the performance of the kits. Our study highlights the need for prospective serology studies and good laboratory quality assurance to better understand the humoral response to SARS-CoV-2 infection.

The weakness of the study should be noted that the clinical relevance of antibody titers in protecting against subsequent SARS-CoV-2 infection is uncertain. Compared to ELISAs, neutralization assays require virus culture, are much more labor-intensive, and need to be conducted in laboratories with higher biosafety levels [15, 16]. We are currently conducting neutralization studies to further investigate whether ELISA results were correlated with neutralization results so far as to substitute for the neutralization test in resource-limited situations.

Although a favorable outcome was achieved in one patient after CP transfusion, the efficacy of CP remains inconclusive due to the very small sample size and other concomitant treatments, which might confound the result.

In summary, we presented serologic findings from six CP donors recovered from COVID-19 and one case treated with CP. This report may help facilitate understanding of the SARS-CoV-2 infection and establish donor screening protocol for CP infusion therapeutics in the COVID-19 outbreak.

## MATERIALS AND METHODS

### Study design

Under the first and second edition of CP infusion therapeutics guidelines approved by the National Health Commission, we developed a protocol for donor screening, plasma collection and specimen analysis to screen potential donors and collect high-titer plasma. Donor screening, specimen collecting and convalescent plasma collecting were conducted at the Second Hospital of Nanjing, a designated medical institution for COVID-19. The antibody testing was conducted in Nanjing Red Cross Blood Center, and its Department of Laboratory Medicine is accredited by China National Accreditation Service for Conformity Assessment. This study was approved by the ethics committee of the Second Hospital of Nanjing (reference number: 2020-LS-ky003). Written informed consent was obtained from all the donors and the recipient.

### Donor population

We screened potential convalescent plasma donors from patients who were confirmed SARS-CoV-2 infection by PCR and had recovered at least four weeks from symptom onset. A total of eight volunteers were recruited as potential plasma donors for assessment. Two were excluded because of elevated alanine transaminase for one case and unexpected hemoglobin levels for the other case. The remaining six provided written, informed consent to become qualified donors.

### Collection of specimens for antibody levels

Convalescent plasma was collected by apheresis from COVID-19 recovered donors, and specimen for antibody testing were collected from an integrated bypass collection reserved sample bag. Plasma for determination of anti-SARS-CoV-2 IgG antibody levels was collected in EDTA tubes and serum for anti-SARS-CoV-2 IgM antibody levels was collected in tubes with coagulation accelerators. Samples were delivered to Nanjing Red Cross Blood Center immediately after collecting, followed by sample centrifuging and antibody testing.

### Serology tests

Two solid-phase microplate ELISAs were employed, based on the nucleocapsid (N) protein of SARS-CoV-2 (Livzon, Diagnostics Inc., Zhuhai, China).

The first kit was a capture enzyme-linked immunosorbent assay for IgM antibody using horseradish peroxidase (HRP)-labeled SARS-CoV-2 antigens. To reveal IgM, serum samples were diluted 1:100 in dilution buffer and allowed to incubate for 60 min with plates coated by anti-human IgM μ chain. Plates were washed and HRP-labeled antigens were added. After 30 min incubation, unbound components were washed away, following adding of TMB substrate with its buffer. For a further 15 min incubation, stop buffer was added and absorbance values were measured at 450nm and 630nm dual-wavelength using a microplate reader.

The second kit was an indirect enzyme-linked immunosorbent assay designed for IgG antibody. After a formulated 1:20 predilution according to the ELISA manufacturer’s instructions, plasma specimens were serially titrated 1:1, 1:10, 1:20, 1:40, 1:80, 1:160 and 1:320 in microplates by plasma from unexposed donors and added to plates coated with SARS-CoV-2 antigens. Following 60 min incubation at 37°C, plates were washed and incubated with horseradish peroxidase-labeled anti-human IgG secondary antibody. Again, plates were washed following 30 min incubation at 37°C and TMB substrate was added with its buffer. 15 min later, stop buffer was added and absorbance values were measured at 450nm and 630nm dual-wavelength using a microplate reader.

Results were reported as the optical density (OD) ratio, which was calculated as the OD value of the donor’s sample divided by the cutoff OD value. We used cutoff values recommended by the ELISA kit manufacturer: a ratio of <1 was considered negative, and ≥1 was considered positive.

### Statistical methods

All data from measurements were displayed as tables and a histogram.
